# Small-area estimation and analysis of HIV/AIDS indicators for precise geographical targeting of health interventions in Nigeria. a spatial microsimulation approach

**DOI:** 10.1186/s12942-023-00341-8

**Published:** 2023-09-20

**Authors:** Eleojo Oluwaseun Abubakar, Niall Cunningham

**Affiliations:** 1 School of Social and Political Science, University of Edinburgh, Edinburgh, EH8 9LD UK; 2 School of Geography, Politics and Sociology, Newcastle University, Newcastle upon Tyne, NE1 7RU UK

**Keywords:** Small-Area estimation, Sexual behaviour, HIV, AIDS, Spatial microsimulation, Geographic targeting, Nigeria

## Abstract

**Background:**

Precise geographical targeting is well recognised as an indispensable intervention strategy for achieving many Sustainable Development Goals (SDGs). This is more cogent for health-related goals such as the reduction of the HIV/AIDS pandemic, which exhibits substantial spatial heterogeneity at various spatial scales (including at microscale levels). Despite the dire data limitations in Low and Middle Income Countries (LMICs), it is essential to produce fine-scale estimates of health-related indicators such as HIV/AIDS. Existing small-area estimates (SAEs) incorporate limited synthesis of the spatial and socio-behavioural aspects of the HIV/AIDS pandemic and/or are not adequately grounded in international indicator frameworks for sustainable development initiatives. They are, therefore, of limited policy-relevance, not least because of their inability to provide necessary fine-scale socio-spatial disaggregation of relevant indicators.

**Methods:**

The current study attempts to overcome these challenges through innovative utilisation of gridded demographic datasets for SAEs as well as the mapping of standard HIV/AIDS indicators in LMICs using spatial microsimulation (SMS).

**Results:**

The result is a spatially enriched synthetic individual-level population of the study area as well as microscale estimates of four standard HIV/AIDS and sexual behaviour indicators. The analysis of these indicators follows similar studies with the added advantage of mapping fine-grained spatial patterns to facilitate precise geographical targeting of relevant interventions. In doing so, the need to explicate socio-spatial variations through proper socioeconomic disaggregation of data is reiterated.

**Conclusions:**

In addition to creating SAEs of standard health-related indicators from disparate multivariate data, the outputs make it possible to establish more robust links (even at individual levels) with other mesoscale models, thereby enabling spatial analytics to be more responsive to evidence-based policymaking in LMICs. It is hoped that international organisations concerned with producing SDG-related indicators for LMICs move towards SAEs of such metrics using methods like SMS.

## Introduction

The intensity of the HIV/AIDS pandemic, its key drivers, and the outcomes of public interventions all exhibit significant spatial heterogeneity, not only at large regional scales, but also at microscale levels like towns, communities and neighbourhoods [1]. This motivates the overwhelming emphasis on precise geographical targeting by several authors and organisations, including the Joint United Nations Programme on HIV/AIDS (UNAIDS) [2–4]. The lack of small-area estimates (SAEs) of standard HIV-related indicators in LMICs greatly undermines the possibility of tracking trends and patterns of HIV-related metrics at fine spatial scales, as is typically done for large regions like countries [5]. This is particularly problematic in LMICs like Nigeria, which suffer from a scarcity of geocoded data amidst the high prevalence rate of HIV/AIDS. A clear implication is that regardless of overall records of positive outcomes for large regions, the localities therein (particularly disadvantaged ones) not only may be suffering a much slower pace of improvement, but also could be experiencing deteriorating outcomes. In addition to greatly hampering the spatial allocative efficiency of public resources for the control of the HIV/AIDS pandemic, the dearth of fine-scale metrics also limits the possibility of determining contextual drivers of these indicators. Consequently, the differential effects of intervention programmes upon various localities remain obscure. In practical terms, the ability to obtain granular estimates of standard HIV-related indicators in this paper also means that a wide variety of robust spatial analytics can be implemented in LMICs using a realistic spatially enriched synthetic population.

Even though there has been a recent upsurge in the number of studies concerned with fine-scale mapping of HIV-related indicators [6], the vast majority of these works do not adequately synthesise locational and social (population at risk) aspects in a unified framework. This owes largely to limitations regarding the methods employed, which are seldom as robust as the spatial microsimulation (SMS) employed in the present study. Furthermore, SAE of HIV/AIDS indicators are yet to benefit from the immense analytical power of SMS, which typically requires rich data that are scarce in LMICs [7, 8]. Nevertheless, this has been employed in the analysis of other health phenomena, including smoking, obesity, mental illness, alcohol consumption, diabetes, and healthcare access, especially in developed countries, which do not experience the same data limitations as in the present study context [10–15]. Similar indicators and data have been used by other studies, except that these are not disaggregated at small-area scales like in the present study [16–20]. Mweemba et al. [5] is a recent similar attempt at estimating the HIV prevalence in Africa (Zambia); however, the SAE method employed is not as robust as SMS. Overall, many of these studies are not based on standard SDG-related estimates such as the Multiple Indicator Cluster Survey (MICS), Millennium Development Goals (MDG) or Sustainable Development Goals (SDG) Indicators framework [21–23]. This is a major limitation to their reproducibility and comparison in different international contexts as well as their utility for the monitoring of sustainable development initiatives.

To advance ongoing research in this area, there are two (2) main objectives of this paper. The first objective is to simulate a spatially enriched synthetic population by linking survey microdata with small-area zoning systems using SMS. The second is to utilise the derived spatially enriched synthetic individual-level population to estimate and analyse four SDG-related multivariate indicators of HIV/AIDS at small-area levels. These indicators are (1) sex with non-regular partners (MICS Indicator 9.14), (2) condom use with non-regular partners (MICS Indicator 9.15 or MDG Indicator 6.2), (3) knowledge about HIV prevention among young persons (MICS Indicator 9.1 or MDG Indicator 6.3) and (4) sexually active young persons who have been tested for HIV and know the results (MICS Indicator 9.6). In the MICS indicators framework, these are amongst the 17 sub-indicators which capture traits of HIV/AIDS and sexual behaviour. These standard multivariate MDG or SDG indicators of HIV/AIDS and sexual behaviour are estimated and analysed for small areas with a view to facilitating precise geographical targeting of HIV-related interventions [1, 24–26]. Not only is the estimation of HIV/AIDS and sexual behaviour indicators in this study premised on the framework of social determinants of sexual behaviour and sexual health [25, 75], these are also amongst the internationally adopted indicators for monitoring HIV/AIDS the world over.

## Methods

The MICS indicators framework which is congruent with relevant SDG indicators, is operationalised in this study using the fifth wave of MICS microdata collected by UNICEF in 2016/2017. This also has the benefit of temporality and comparability for reproducing similar research both with historical MICS data (since the 1990s) and for the 118 countries currently being covered. About 130 indicators are collected by MICS to analyse the situation of children, women and men in the areas of health, education and child protection. With roughly 33 SDG indicators, MICS is a rich data source for analysing aspects of the SDG including HIV/AIDS of this study. At the time of writing, the fifth wave was the latest MICS microdata of the study area available for public download on UNICEF’s website (https://mics.unicef.org/surveys). There are indications that MICS 5 is representative at the subnational levels like the study area, Kogi State (see https://mics.unicef.org/faq#sampling). This explains why the official MICS 5 report of Nigeria disaggregates several statistics at the state level [27]. If subnational units of the MICS data were not representative, we suppose they would not be appropriate grouping/clustering levels for use in multilevel spatial analysis [28] or other types of sub-national spatial analysis [29, 30]. Aside from these, a cardinal objective of SMS is to mitigate the problem of data sparsity (of sample surveys) through data enrichment by synthesizing them with spatial constraints that contain the entire study population in aggregate form. Thus, sample size limitations of surveys are of minimal concern for this type of analysis since this is the challenge that SMS is meant to overcome.

**Fig. 1:**
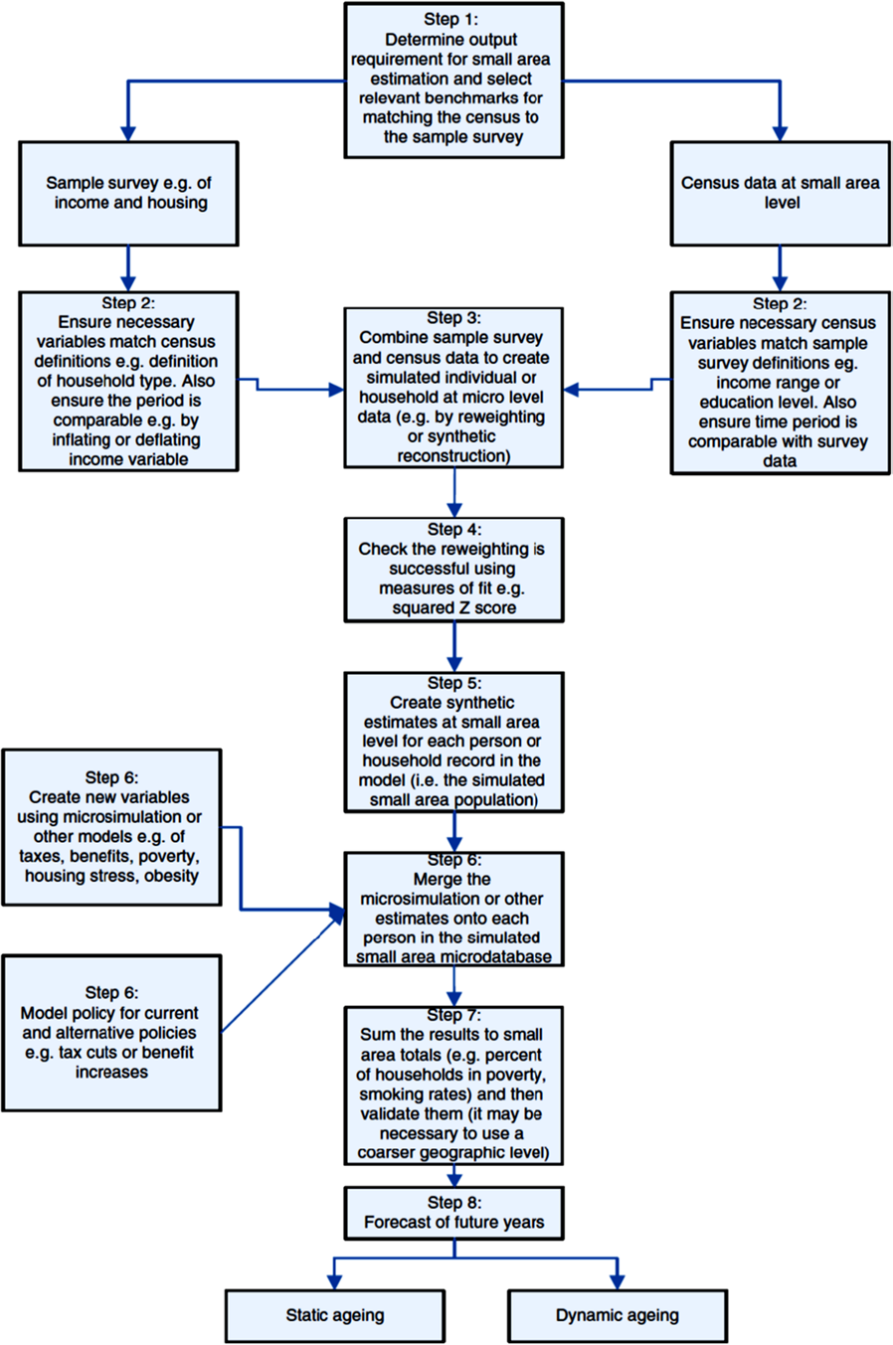
Overview of steps required to construct a spatial microsimulation model (Clarke and Harding, 2013, p. 262)

As illustrated in Fig. 1, this study follows the standard SMS procedure for developing a spatially enriched synthetic population and deriving SAEs of relevant multivariate outcomes such as health-relevant indicators [31]. ArcGIS Desktop 10.5 and IBM SPSS v26 were used for preparing the microdata and spatial constraints, while Flexible Modelling Framework (FMF) v1.3 [32, 33] was used to simulate a generalised synthetic micro-population of the study area, which was then used for SAEs of relevant MICS 5 indicators. FMF software is mature SMS software [34] as well as the most user-friendly of the available options. This implements the combinatorial optimization approach which has been adjudged to outperform many competing techniques, including Iterative Proportional Fitting (IPF) and Generalised Regression Weighting (GREGWT) [35, 36]. Other specialist software for implementing SMS include LIAM2 [37], the SMS package of R [38] and JAS-mine [39].

### Selecting and preparing spatial constraints

The selection of appropriate spatial constraints can be determined via regression analysis, analysis of intra-area homogeneity, literature reviews, and consultation with end users [36, 40, 41]. Where the outcomes of an SMS operation are multivariate, such as in the present study, it is essential to include key demographic attributes as spatial constraints, especially in equity-sensitive projects [42]. This can be informed by the framework of PROGRESS stratifiers, which represents the internationally adopted standard for statistical data disaggregation in SDG-related studies [43, 44]. However, the availability and quality of relevant datasets for the study area also limits the selection of variables for this study.

All of the spatial constraints and sub-constraints in this study were derived from the following WorldPop grids: age/sex structure, education (per sex) (as illiteracy ratio) and poverty. From the age/sex structure grid, three (3) spatial constraints were produced, namely: age [5 year groupings], sex (female or male), and 5 year age groupings by sex. From the education grid, two (2) spatial constraints were derived, namely: education (educated or uneducated) and education by sex (i.e., educated female, uneducated female, educated male or uneducated male). The poverty grid produced one spatial constraint, poverty (i.e. poor or not poor). As required, these were aggregated to the small-area analytical zoning system of the study area using ArcGIS Desktop 10.5 software. The configuration of the variables used for linking the microdata with the spatial constraints of the SMS operation is outlined in Table 1.

**Table 1:** The spatial constraints used in the SMS of Kogi State synthetic population (of persons age 15–49 years)

Spatial constraint	Sub-constraints	
Age (years)		
	15–19	35–39
	20–24	40–44
	25–29	45–49
	30–34	
Sex		
	Female	Male
Education		
	Educated	Uneducated
Poverty		
	Poor	Not Poor
Sex by education		
	Female educated	Male educated
	Female uneducated	Male uneducated
Sex by age		
	Female 15–19	Male 15–19
	Female 20–24	Male 20–24
	Female 25–29	Male 25–29
	Female 30–34	Male 30–34
	Female 35–39	Male 35–39
	Female 40–44	Male 40–44
	Female 45–49	Male 45–49

Most processes of data preparation for SMS involve the categorisation of continuous variables or the recoding of variables into fewer classes. Though necessary for model execution, error mitigation, and model validity, data preparation often results in a loss of nuance and a consequent reduction in the robustness of the derived synthetic population.

### Preparing the microdata/population

Only respondents from Kogi State are included in the microdata, although SMS allows the use of microdata from a wider geographical area (and collected at different times) than that which is being modelled [45, 46]. Relevant variables of the microdata were recoded accordingly to match the configuration of the available spatial constraints. For instance, since only two categories of socioeconomic status are provided by spatial constraints (namely Poor and Not Poor), the wealth index quintile of the microdata was regrouped into two corresponding categories. ‘Poorest’ (coded 1) and ‘Second’ (coded 2) were regrouped as ‘Poor’, while ‘Middle’ (coded 3), ‘Fourth’ (coded 4) and ‘Richest’ (coded 5) were regrouped as ‘Not Poor’. Similarly, the multiple levels of education present in the MICS 5 microdata were regrouped into two categories, namely ‘Educated’ and ‘Uneducated’, to match available spatial constraints. The selected variables and structure of recoded microdata used as linkage variables are outlined in Table 1. Only respondents aged 15–49 years with all of the relevant linking variables are included in the SMS operation in order to minimise modelling errors. Thus, the input MICS 5 microdata contain 1305 persons, comprising 912 females and 393 males.

### Implementing the SMS of Kogi state population

SMS requires two (2) types of appropriately structured data input, namely: spatial constraint files and a microdata/population file. With these, the SMS operation in this paper was performed using FMF v1.3 software, following the detailed user guide of Harland [32]. The same process can also be implemented in R following the comprehensive guidelines by Lovelace and Dumont [34].

The execution of the SMS within the FMF software environment is a computing-intensive process which produced a spatially enriched synthetic population of the study area, as expected. The synthetic small-area analytical zone to which each synthetic person belongs is also included as a column of the resulting table. These enabled the computation and mapping of SAEs of four (4) MICS 5 indicators of HIV/AIDS for young people aged 15–24 years in the study area. These are (1) comprehensive knowledge about HIV prevention (MICS 5 Indicator 9.1 or MDG Indicator 6.3), (2) sexually active young persons who have been tested for HIV and know the results (MICS 5 Indicator 9.6), (3) sex with non-regular partners in the last 12 months (MICS 5 Indicator 9.14), and (4) condom use with non-regular partners (MICS 5 Indicator 9.15).

The computation of these indicator estimates followed the standard MICS 5 indicator computation algorithms but was adapted so as to account for the new small-area geographies ascribed to the synthetic individual-level population. Furthermore, other relevant disaggregation variables not considered in the original algorithms were also included, particularly the ethnicity and religion of household heads. This entailed making requisite modifications to the standard MICS 5 indicator computation algorithms provided as SPSS syntax files (sps) on the MICS 5 website: http://mics.unicef.org/tools?round=mics5#data-processing. The goodness of fit of the derived synthetic population is examined in the next section, after which the SAEs derived thereof are discussed.

## Results

### The spatially enriched synthetic micro-population

The result of the SMS operation in this study is a spatially enriched synthetic individual-level population of the study area comprising 2,249,170 microunits (i.e. persons of age 15–49 years). These are composed of 1,115,283 females and 1,133,887 males, with about 425 MICS 5 attributes. The synthetic small-area analytical zone to which each synthetic person belongs is also included as a column of the resulting table. These data are available at [47]. With the derived spatially enriched synthetic population, it became possible to compute and map SAEs (of relevant MICS 5 indicators) for the optimised analytical zoning system of the study area.

### Goodness of fit of the synthetic population: internal validation

It is crucial for a derived synthetic population to be validated before being utilised for further analytical steps [48]. Not only does this ensure that a given SMS is of acceptable quality, it also ensures that potential analytical errors are not propagated unto further analytical operations. This explains why Step 4 of Fig. 1, which is the validation of a synthetic population, precedes ‘the creation of estimates at small-area levels’ (i.e. Step 5) as well as subsequent analytical steps in SMS. Internal validation is performed in this study by comparing the outputs of the SMS (i.e. the synthetic population) with the original inputs (i.e. the sample survey micro-population and the spatial constraints) to estimate the extent to which the original inputs are emulated by the resulting synthetic population [49, 50]. The degree of modelling accuracy is assessed using all twelve [12] goodness-of-fit statistics available in FMF software, namely: standard root mean square error (SRMSE), absolute entropy difference (AED), R-squared (R^2^), entropy (standard entropy measure), chi-squared (X^2^), total absolute error (TAE), standard absolute error (SAE), percentage error (PE), total error (TE), cell percentage error (CPE), Z^2^, and Z.

**Fig. 2:**
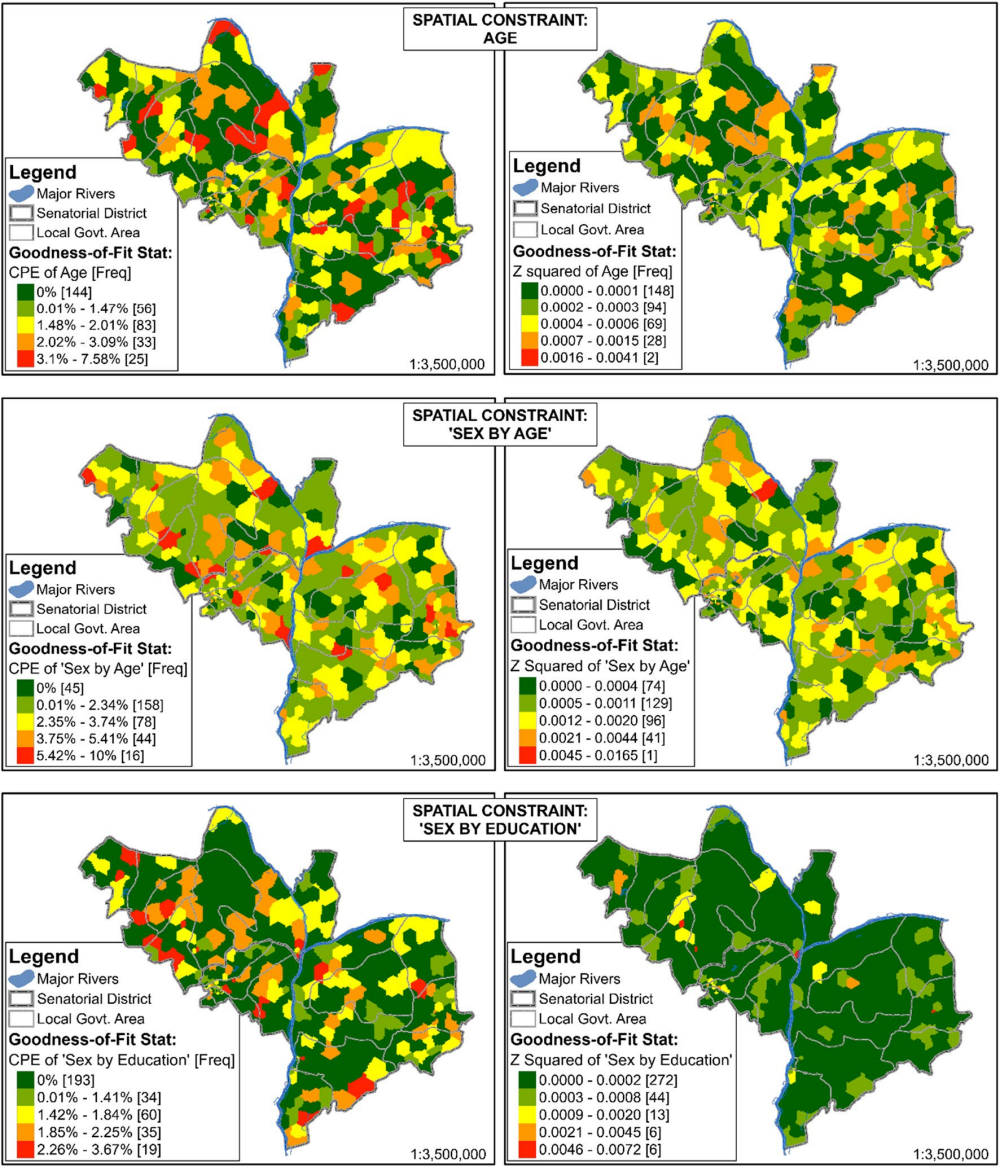
Some Goodness-of-Fit statistics for three of the constraints used for the SMS of Kogi State, mapped for each synthetic small-area zone of the study area. The greener, the more accurate, while with increasing redness comes increasing relative zonal error

These goodness-of-fit statistics are produced for each spatial constraint and small-area zone, constituting a massive table of 342 rows by 73 columns. Presented in are the goodness-of-fit statistics for each of the spatial constraints considered, while Fig. 2 presents maps of two of these goodness-of-fit statistics (i.e. CPE and Z^2^) for three spatial constraints, namely: ‘age’, ‘age by sex’, and ‘sex by education’. Z-squared is chosen for mapping because of its popularity in cognate literature [51, 52], while CPE is relatively easy to explain. With higher values of SRMSE, AED, SAE, PE and CPE come more error in a model fit. The three spatial constraints mapped tend to have the lowest goodness-of-fit values, as shown in Table 2 (also highlighted in orange colour); therefore, they deserve further consideration. Nevertheless, these error metrics are extremely low, being less than 0.08. This implies very good internal validity of the derived synthetic micro-population of Kogi State.

**Table 2:** Various goodness-of-fit statistics for the validation of the SMS of Kogi State

Variable Name	SRMSE	AED	R^2^	Entropy	Chi^2^	TAE	SAE	PE	TE	CPE	Z^2^	Z
Sex	2.84E− 05	4.02E− 08	1	6.499267	0.002023	6	2.67E− 06	1.33E− 04	3	2.67E− 04	5.06E− 04	0
Age	3.40E− 04	7.98E− 06	0.999999	7.671999	0.323618	243	1.08E− 04	0.005402	121.5	0.010804	0.08323	0
Sex by Age	6.99E− 04	4.82E− 06	0.999998	8.361252	1.429437	513	2.28E− 04	0.011404	256.5	0.022809	0.367522	0
Education	1.24E− 04	7.79E− 06	1	6.331046	0.061976	114	5.07E− 05	0.002534	57	0.005069	0.015504	0
Sex by Education	2.17E− 04	3.27E− 06	1	6.973711	0	175	7.78E− 05	0.00389	87.5	0.007781	0.098679	0
Poverty	1.26E− 04	4.35E− 06	1	6.468207	0.041353	118	5.25E− 05	0.002623	59	0.005246	0.010353	− 0.01036

Overall, from the computed goodness-of-fit statistics, the SMS of Kogi State shows an excellent level of accuracy across all of the spatial constraints utilised. For instance, all SAE (and TAE and TE) values are extremely low, with ‘sex’ exhibiting the best model fit and ‘sex by age’ having the lowest (albeit a negligible) model fit. It is unsurprising that ‘sex’ has the best model fit because it was specified as the reference spatial constraint used by FMF software for determining zone totals; thus, it is the reference benchmark by which the accuracy of the synthetic population in this study is adjudged. Any constraint can be chosen as the reference variable for internal validation; however, sex was chosen because it is bivariate and tends to constitute relatively accurate input data. This near-perfect SMS validity is exhibited by all of the other computed goodness-of-fit statistics. For instance, R^2^ values (i.e. coefficient of determination) typically range from 0 to 1, with 1 signifying a perfect model fit. Notice that the R^2^ for all of the constraint variables is approximately 1. Furthermore, a synthetic population is deemed to be unfit if the Z-squared statistic is greater than the critical value (i.e. |Z|> 1.96) [53]. Clearly, no Z^2^ is above the critical value for the current SMS operation, thus confirming the validity of the synthetic population developed in this study.

Figure 2 shows the spatial patterns of CPE and Z-squared for three spatial constraints, namely: ‘age’, ‘age by sex’ and ‘age by education’. These three spatial constraints tend to have the lowest goodness-of-fit values; therefore, they are likely to reveal problem small areas as well as to show substantial spatial variations in mapped data of SMS errors. Like Table 2, Fig. 2 shows that ‘Age by Sex’ has the highest error of any small-area zone, with 16 zones having CPEs of between 5.42% and 10%. Age has the second-highest error, with 25 zones having CPEs of between 3.1% and 7.58%. Nevertheless, not only are these CPE values relatively low, only very few zones have these levels of error, that is, 16, 25 and 19 zones (out of the 341 analytical small-area zones in the study area), for the ‘sex by age’, ‘age’, and ‘sex by education’ spatial constraints, respectively. On the flipside, 45, 144 and 193 zones are 100% accurate in terms of CPE for the ‘Sex by Age’, ‘Age’, and ‘Sex by Education’ spatial constraints, respectively. These corroborate Table 2 in demonstrating the very high internal validity of the SMS in the current study. The resulting synthetic population is, therefore, fit for use in subsequent sections of this paper.

### Small-area estimates of select HIV/AIDS indicators

From the derived synthetic micro-population of the study area, the four (4) SAEs of standard MICS 5 indicators are presented in this section as a prelude to the associated discussion in the subsequent section.

**Fig. 3:**
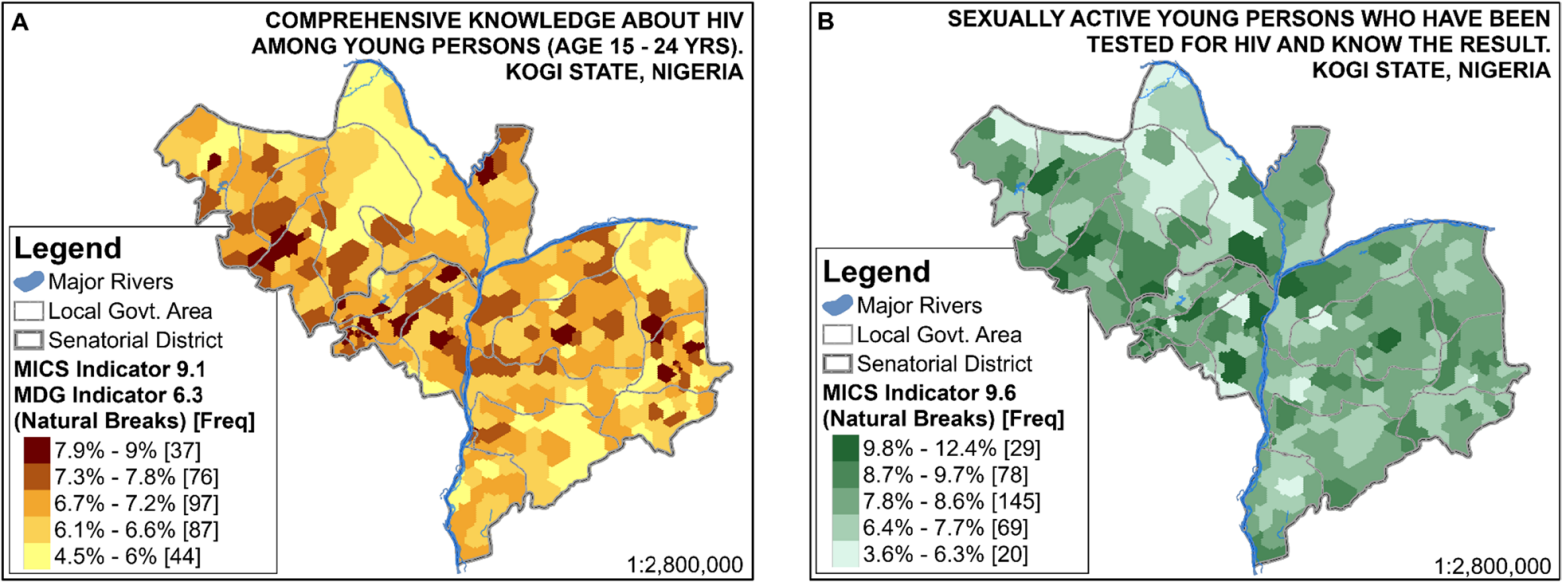
Small-area estimates of MICS 5 Indicators 9.1 and 9.6

**Fig. 4:**
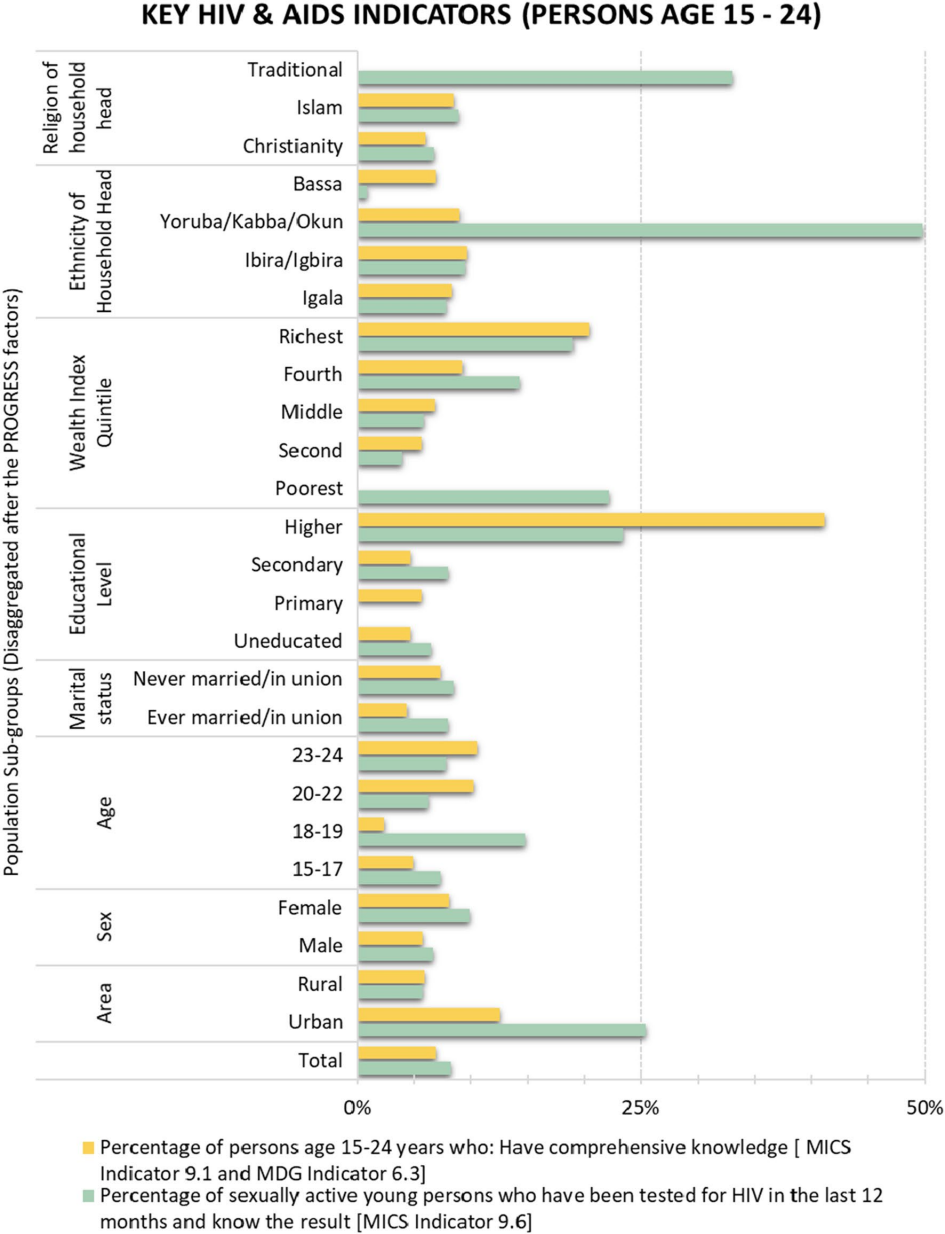
Small-area estimates of MICS 5 Indicators 9.1 and 9.6 disaggregated by key PROGRESS factors

The spatial pattern of comprehensive HIV/AIDS knowledge among young persons aged 15–24 years (MICS 5 Indicator 9.1 and MDG Indicator 6.3) shown in Fig. 3A suggests that there are a relatively greater number of young persons within the middle horizontal strip of the study area who have comprehensive knowledge about HIV than of people in the upper and lower flanks. The small-area spatial pattern of HIV testing amongst sexually active young persons (MICS 5 Indicator 9.6) is shown in Fig. 3B. This suggests that there is more HIV testing amongst sexually active young persons on the western side of the River Niger (and more variations in the same region). The percentage of young persons who have tested for HIV in the last 12 months and know the results ranges from 3.6% to 12.4% in the study area. MICS 5 Indicators 9.1 and 9.6 are further disaggregated socioeconomically for the study area, as presented in Fig. 4. This shows that more young people in urban areas have comprehensive knowledge of HIV (about 12.6%) than have those in rural areas (about 5.9%). Similarly, in the last 12 months, there has been much more HIV testing amongst sexually active young persons in urban areas (about 25.4%) in comparison to rural areas (about 5.8%).

**Fig. 5:**
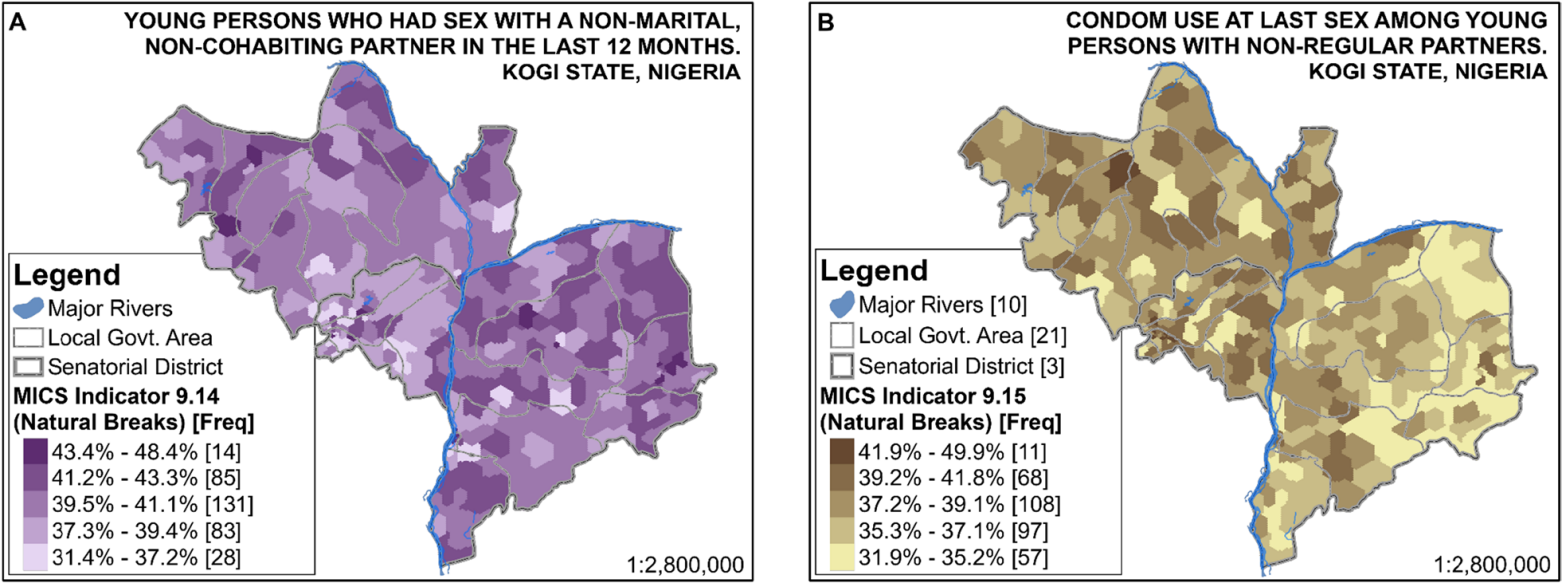
Small-Area Estimates of MICS 5 Indicators 9.14 and 9.15

The percentage of multiple sexual partnerships among young people in Kogi State (MICS 5 Indicator 9.14) ranges from 31.4% to 48.4%, whereas 31.9% to 49.9% of this population had used a condom during their last sexual intercourse with a non-regular partner (i.e. MICS 5 Indicator 9.15). This is shown in Fig. 5A, B respectively. These are further disaggregated according to relevant socioeconomic markers, as shown inFig. 6,^1^ There is an indication that with higher levels of education come increased proportions of young people reporting having had sex with multiple sexual partners in the last 12 months. For instance, 64.9% of young people with higher education reported having had sex with a non-regular partner in the last 12 months, compared to 29.9% of young people with primary education reporting a similar practice. The disaggregation of MICS 5 Indicators 9.14 and 9.15 by marital status, sex, and urban/rural dwelling reveals other interesting patterns which are discussed in the next section.

**Fig. 6:**
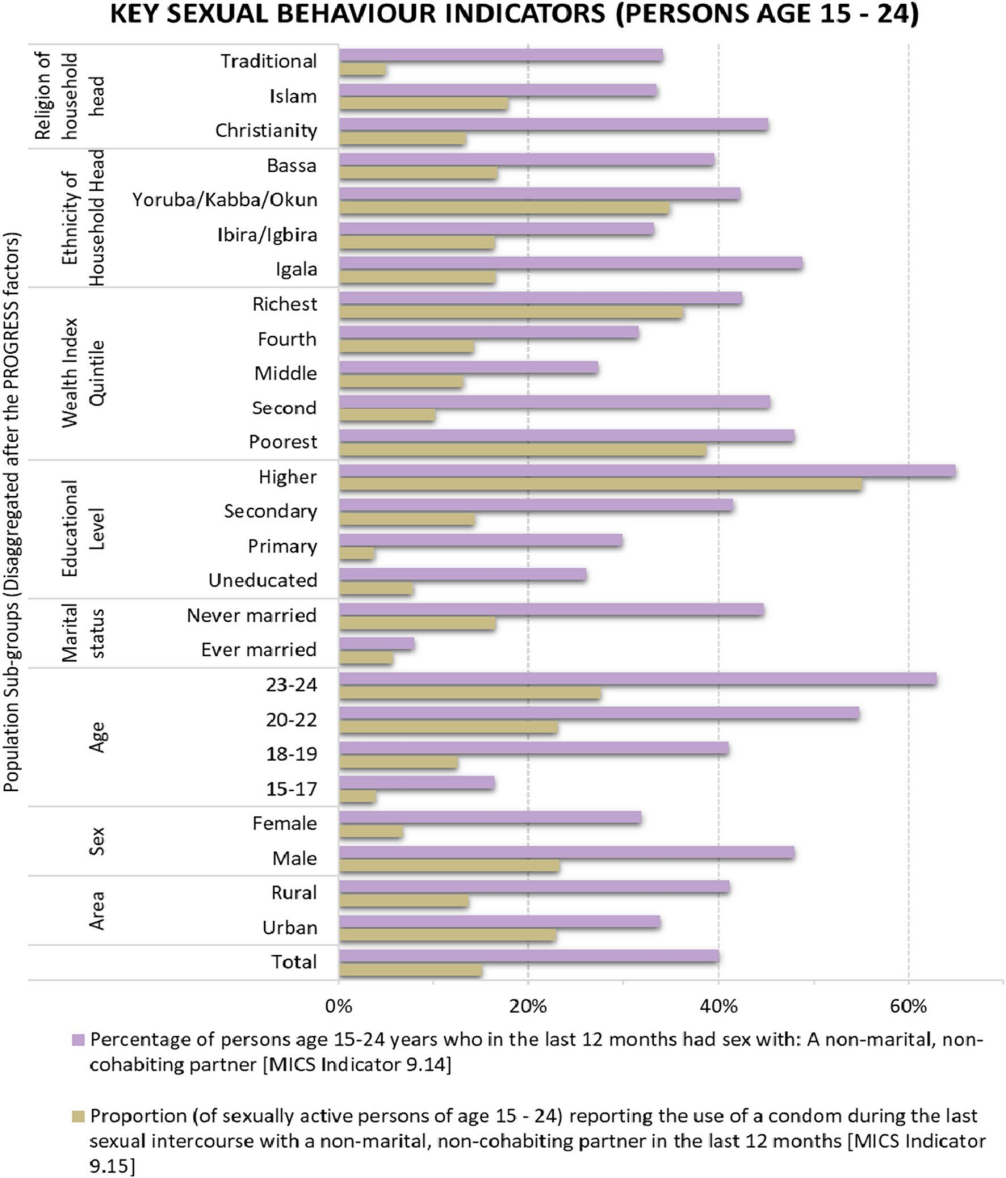
Small-Area Estimates of MICS 5 Indicators 9.14 and 9.15 disaggregated by key PROGRESS factors

## Discussion

### HIV/AIDS knowledge and testing amongst young people (MICS 5 Indicators 9.1 and 9.6)

It is not certain as to why there are a relatively greater number of young persons within the middle horizontal strip of the study area who have comprehensive knowledge about HIV (MICS 5 Indicator 9.1 and MDG Indicator 6.3) than of people in the northern and southern flanks. However, better knowledge about HIV is likely to be associated with both urban dwelling and more education [54, 55]. More educational opportunities as well as urban dwelling tend to be concentrated in the same middle horizontal strip of the study area. Nevertheless, the percentage of young persons with comprehensive HIV knowledge in the study area is generally low, ranging from 4 to 9%. This implies a need to improve HIV-related education in the study area, particularly in the northern and southern flanks.

From the mapping of SAEs of MICS 5 Indicator 9.6 shown in Fig. 3B, not only is the percentage of young persons who have tested for HIV in the last 12 months and know the results very low (3.6% to 12.4%), places with exceptionally poor testing for HIV are also revealed. This indicates that while the study area will benefit from universal improvement in the coverage of HIV testing for sexually active young persons, localities with exceptionally low records of HIV testing (3.6% to 6.3%) should be targeted for prioritised provision of HIV testing services as well as awareness creation [1].

The suggested urban advantage (shown in Fig. 3) is plausible for a number of reasons, including relatively higher health literacy and better accessibility of HIV services in comparison to rural areas [56, 57]. This indicates that rural areas should be given prioritised attention for HIV/AIDS-related interventions. Furthermore, higher levels of both education and wealth quintiles are associated with remarkable increases in both Indicators 9.1 and 9.6. Together with urban residence, wealth and education are key markers of socioeconomic advantage. As these have been shown to be greatly associated with better knowledge of, as well as more testing for, HIV amongst young people, poor knowledge of HIV and a lack of HIV testing amongst sexually active young people can be considered matters of disadvantage in respect of socioeconomic status. This supports studies which show that even though highly educated and/or well-off people are more likely to engage in risky sexual behaviours (such as keeping multiple sexual partners), they also tend to both be more health-literate and to practise safe sex, thereby being less prone to HIV infection than are poor and/or less educated people [54, 55, 58]. Theoretically, there is a complex relationship between risky sexual behaviours and both schooling and wealth, which also varies by gender [16]. Nevertheless, the spatial and social disaggregation of these indicators suggests socio-spatial inequalities that are worth investigating further in subsequent studies.

### Multiple sexual partnerships and condom use amongst young people (MICS 5 Indicators 9.14 and 9.15)

Small-area estimates of MICS 5 Indicators 9.14 and 9.15 (shown in Fig. 5A, B respectively) indicate that even though a large proportion of the young population in the study area had had multiple sexual partners in the last 12 months, a good percentage of these had used a condom during their last sexual intercourse with a non-regular partner. However, the observed spatial patterns in Fig. 5 suggest an inverse relationship between multiple sexual partnerships and condom use amongst young persons in the study area. For instance, relative to other senatorial districts, a smaller proportion of young persons in Kogi Central have multiple sexual partners, while a greater percentage of them reported having used a condom during their last sexual intercourse with a non-regular partner. Conversely, Kogi East, which records a high proportion of multiple sexual partnerships among young people, is also the senatorial district with a relatively low proportion of condom use during their last sexual intercourse with a non-regular partner. This shows that although there is a need to improve sex education and HIV/AIDS-related healthcare/screening services in the study area, this is particularly more crucial in Kogi East to ensure that the higher proportion of multiple sexual partnerships are matched with safe-sex practices.

Despite the increased sexual partnerships associated with higher levels of education depicted in Fig. 5, these are matched with more reports of regular usage of condoms [56, 58]. In fact, 84.8% of this population had used a condom during their last sexual affair with a non-regular partner, compared to 12.6% of young people with primary education who reported having used a condom during a similar sexual encounter. This shows that although more educated young people tend to be more exploratory regarding having sex with multiple sexual partners, they are also more cautious in practising safe sex (by using a condom with non-regular partners) than are their less educated peers [57, 59]. This suggests a need for more sex education for young people with secondary education or lower. For young people aged 15 – 24 years, a key reason for increased multiple sexual partnerships with higher education is that time in higher education and beyond is often associated with more liberty/autonomy, being away from the restrictions of parents or guardians [60, 61]. Apparently, the educational level of young people is likely to be directly correlated with age. Results show that with increased age comes more tendency towards sex with multiple sexual partners amongst young people; however, a greater proportion of non-teenage young people (42.1% and 43.8% for ages 20–22 and 23–24 respectively) reported having used a condom during their last sexual affair with a non-regular partner in comparison to the proportion of teenage young people (23.5% and 30.6% for ages 15–17 and 18–19 respectively) reporting the usage of condoms. This is consistent with extant empirical literature, which suggests that awareness of safe-sex practices is positively related to age, education, and socioeconomic status [62]. As with having a higher level of education, post-teenage young people are likely to be more autonomous than teenage young people, since they may no longer be subject to as many parental restrictions as those imposed on teenagers [60, 61]. Despite this, they tend to be twice as likely to practise safe sex as teenage young people. Thus, teenage young people should be prioritised for HIV-related interventions, especially on the need for safer sexual behaviour.

Other interesting patterns are also observable when Indicators 9.14 and 9.15 are disaggregated according to marital status, sex, and urban/rural dwelling. Being in non-committal relationships, many more single young people (44.7%) have had sex with multiple sexual partners in the last 12 months in comparison to their married peers (7.9%), as expected [63, 64]. Furthermore, amongst married young people who have had an extramarital sexual partner in the last 12 months, the large majority (72.2%) reported having used a condom, compared to the proportion of single young people who reported having used a condom with their non-regular sexual partner in the same period (36.8%). For cultural and religious reasons, married young people in the study area are much less likely to engage in extramarital sex than are their single peers [65]. Moreover, having a married regular sexual partner means that most married young people are not likely to be keenly searching for new sexual partners. Whenever such extramarital affairs happen, condoms are often used to prevent both pregnancy of the non-regular (female) partner as well as the transmission of an STI/STD to their married spouse.

It is unsurprising that a greater proportion of young males have had sex with a non-regular sexual partner (48.0%) in comparison to the proportion of females with multiple sexual partners in the last 12 months (31.9%) [66–68]. This is because Nigeria, like many sub-Saharan African countries, is notoriously patriarchal, thus being culpable of hegemonic masculinity [69–71]. This concept develops on ideas of patriarchy in explaining entrenched patterns of social practices (including actions and expectations) that perpetuate male dominance of females, often facilitated by culture, institutions, and political influence [72]. Consequently, while females are highly discouraged from keeping multiple sexual partners for cultural reasons, males do not experience the same levels of restrictions, even when married [73, 74]. Indeed, in many localities (including the study area), while it is taboo for married females to engage in extramarital sex, this is not the case for married males. It is ironic, however, that a higher proportion of married young males who reported having had sex with a non-regular partner in the last 12 months (48.4%) indicated having used a condom in such affairs, compared to the percentage of their female peers who reported having used a condom during their last sexual affair with a non-regular partner (21.0%) [75–77]. This may be because in the study area there is a tendency for (married) females who have sex with non-regular partners to do so for a variety of transactional reasons (including in exchange for gifts or other favours from men), in which case they are less able to negotiate for safe sex [78–80]. Consequently, young males are twice as likely to practise safe sex with a non-regular partner as females, thereby partly explaining why young females (aged 15–24 years) in sub-Saharan Africa are twice as likely as young males to have HIV [81]. This suggests a need to promote safer-sex practices amongst young people, especially females, as well as to target empowerment interventions at women to make them less vulnerable [16].

In rural areas, more young people reported having had sex with a non-regular partner (41.1%) in the last 12 months, compared to their peers in urban areas who reported a similar practice (33.9%) [82]. This suggests that young people in rural areas enjoy more autonomy than do their peers in urban centres, probably because of the very informal and communal nature of rural areas in the study area. With this comes an increased risk of HIV infection because people who become sexually active at a younger age are more prone to having multiple sexual partners in their lifetime, which is associated with a higher tendency towards indulging in other risky sexual behaviours [58, 63]. This is, however, slightly at odds with the patterns recorded based on disaggregation by educational level, as a greater proportion of young people in urban areas are expected to possess higher education in comparison to their peers in rural areas. Despite more reports of having had sex with non-regular partners amongst young people in rural areas, a much lower proportion of these people use condoms in comparison to their peers in urban areas [82]. While 67.5% of young people with multiple sexual partners in urban areas had used a condom during their last sexual intercourse with a non-regular partner, only 33.3% of young people in rural areas reported the usage of condoms. Thus, young rural dwellers are twice as unlikely to practise safe sex with a non-regular partner than their urban peers. In addition to poorer access to condoms in rural areas, this could indicate lower levels of sex education and, by extension, less awareness of safe-sex practices than in urban areas [54, 57]. On the one hand, this suggests a need for increased sex education, condom accessibility, and the promotion of safe-sex practices in rural areas. On the other hand, being more vulnerable, rural areas should be targeted for increased accessibility to medical services related to the sexual health of young people, such as HIV/AIDS-related services as well as services for other STDs/STIs, such as relevant screenings/tests [1, 83].

## Conclusion

Unlike purely statistical methods, SMS is arguably the only explicitly geographical method for SAEs of multivariate indicators, especially when there is a necessity to simultaneously incorporate multiple variables [84]. From MICS 5 microdata, four (4) standard indicators that relate to HIV/AIDS and sexual behaviour are estimated (and mapped) at a small-area scale and comprehensively analysed. In tandem with a need to illuminate inequalities using the PROGRESS framework [44], these indicators are also disaggregated according to relevant socioeconomic dimensions in order to explicate pertinent socio-spatial variations [85]. In addition to creating SAEs of standard health-related indicators from disparate multivariate data, the outputs make it possible to establish more robust links (even at individual levels) with other mesoscale models, thereby enabling spatial analytics to be more responsive to evidence-based policymaking in LMICs [53, 86–88]. With SMS, it is also possible to further link and co-analyse various other types of contextual information at multiple spatial scales with the rich socioeconomic data of sample surveys. What is more, this study provides a robust analytical framework for estimating and analysing other health—and/or SDG-related indicators at very granular scales in various data-sparce contexts through the use of a variety of publicly available sample survey microdata and gridded demographic datasets.

From anecdotal evidence, spatial patterns with regard to both ethnicity and religion are likely to be unreliable because none of the spatial constraints are known to have a significant correlation with these two in the study area. In other words, sex, age, wealth, and educational level are not known to be significantly correlated with ethnicity or religion in the study area. Future works would benefit from having either ethnicity or religion as a spatial constraint because in the study area any of these would serve as a good predictor of the other. This would go a long way in enhancing the fidelity of the resulting synthetic population in these dimensions. For some of the spatial constraints, such as wealth/poverty and literacy, only a binary categorisation was used instead of the full 4–5 categories in the MICS 5 sample survey microdata. This is a limitation of the gridded demographic data used in this project. New data sources offer opportunities to provide more detailed spatial constraints, which will enhance the model fidelity. For instance, comprehensive data on rural/urban classification are now available from the Global Human Settlement—Settlement Model Grid (GHS-SMOD) [89]. Furthermore, future works should consider using a variety of innovative data sources to implement dynamic SMSs of standard SDG-related indicators beyond the health-related exemplars in the present study. This would benefit from the use of more robust SMS tools in geocomputation environments like Python and R. It is hoped that in the years ahead, the simulation of small-area metrics of SDG-related indicators will become the standard practice of international organisations concerned with data provision in LMICs.

## Data Availability

The datasets generated and/or analysed in this manuscript are available in the Harvard Dataverse repository, https://dataverse.harvard.edu/privateurl.xhtml?token=b47f9178-ce21-428b-94f0-757b2096c2aa

## Footnotes

1 Indicator 9.15 is depicted relative to Indicator 9.14 such that the golden bars have percentages that are independent of the y-axis of the chart, but relative to the purple bars (of indicator 9.14) in order to be more meaningful and aid proper comparisons.
